# Risk Factors and Clinical Outcomes of Glucose-6-Phosphate Dehydrogenase Deficiency in Neonates: A Case-Control Study

**DOI:** 10.7759/cureus.106191

**Published:** 2026-03-31

**Authors:** Umama B Fatima, Husna Yoosuf, Mariam E Mohamed, Arwa Leelawalla, Jayadevan Sreedharan, Osama E Sayed

**Affiliations:** 1 Department of Medicine, Emirates Health Services (EHS), Ras Al Khaimah, ARE; 2 Department of Medicine, Sheikh Khalifa Medical City (SKMC), Abu Dhabi, ARE; 3 Department of Medicine, Emirates Health Services (EHS), Fujairah, ARE; 4 Department of Medicine, Thumbay University Hospital, Ajman, ARE; 5 Department of Epidemiology and Biostatistics, Gulf Medical University, Ajman, ARE; 6 Department of Pediatrics and Neonatology, Thumbay University Hospital, Ajman, ARE

**Keywords:** case-control study, g6pd deficiency, hyperbilirubinemia, neonates, phototherapy

## Abstract

Background

Glucose-6-phosphate dehydrogenase (G6PD) deficiency is a common inherited enzymatic disorder and a major risk factor for neonatal hyperbilirubinemia. This study aims to identify baseline risk factors and evaluate clinical outcomes associated with G6PD deficiency in neonates.

Methods

A case-control study was conducted, including 150 neonates, comprising 50 G6PD-deficient cases and 100 controls. Demographic characteristics, clinical variables, and laboratory parameters were compared between groups. Categorical variables were analyzed using chi-square (χ²) tests, and continuous variables were analyzed using the Mann-Whitney U test. A p-value <0.05 was considered statistically significant.

Results

Significant associations were observed between G6PD deficiency and male sex, ethnicity, family history of G6PD deficiency, prolonged hospital stay, phototherapy requirement, and presence of jaundice (p <0.001). G6PD-deficient neonates had significantly higher total bilirubin levels and lower G6PD enzyme levels compared with controls. No significant associations were found with gestational age, feeding type, mode of delivery, or maternal and fetal complications.

Conclusions

G6PD deficiency was significantly associated with key risk factors like male gender, ethnicity, family history of G6PD, and clinical outcomes, including neonatal hyperbilirubinemia, jaundice, prolonged hospitalization, and increased phototherapy requirement.

## Introduction

Glucose-6-phosphate dehydrogenase (G6PD) deficiency is one of the most common genetic disorders, affecting approximately 400 million people worldwide [1]. The G6PD enzyme is crucial in the pentose phosphate pathway, where it generates nicotinamide adenine dinucleotide phosphate (NADPH) to maintain reduced glutathione, which protects red blood cells from oxidative damage [2]. Erythrocytes are particularly susceptible to reactive oxygen species (ROS) due to their role in oxygen transport and their inability to replace damaged cellular proteins. Inherited deficiencies of G6PD can lead to acute hemolytic anemia during periods of increased ROS production [3]. Although children with G6PD deficiency are typically asymptomatic, exposure to specific triggers such as certain foods, medications, and infections can provoke hemolytic reactions [4]. This reaction may be caused by oxidative stress from substances like fava beans or certain drugs.

A study in the UAE found that the prevalence of G6PD deficiency among the UAE nationals was 7.4%, with 11.6% in males and 3.6% in females. Among non-UAE nationals, the prevalence was 3.8% [5]. Screening for G6PD deficiency and vigilant monitoring of affected infants are essential in neonatal care to prevent kernicterus, a severe and permanent neurological condition.

The WHO recommends screening all infants for G6PD activity in regions with a high prevalence of deficiency. There is a clear gender difference in the prevalence of G6PD deficiency, with men being far more affected than women. This can be explained by the disorder's X-linked inheritance pattern. Research continuously shows that male newborns have a greater likelihood of G6PD deficiency than female neonates. For example, studies conducted in Iran's Ardabil Province found that the prevalence rate was 80% in males and 20% in girls [1]. Similar to this, research conducted in Saudi Arabia and Egypt discovered around a 3:1 male-to-female ratio [2,6]. According to research done in Abu Dhabi, UAE, the prevalence was 3.6% for females and 11.6% for males [5].

Genetic susceptibility and family history are important factors in the development of G6PD deficiency. Inheritable illnesses, such as G6PD deficiency, may become more prevalent in consanguineous marriages, which are more common in some areas. A comprehensive study of G6PD deficiency epidemiology in Arab nations identified consanguineous marriages and a positive family history as important risk factors [7]. The frequency and manifestation of the illness are also influenced by genetic variation across various populations; for example, some ethnic groups are more likely to have particular G6PD variants.

The distribution of G6PD deficiency is strongly impacted by geographic and ethnic differences. G6PD deficiency is more prevalent in regions historically endemic for malaria, likely due to a selective survival advantage; however, this represents a geographic association rather than a direct causal relationship. For example, studies conducted in Mauritania and Nigeria revealed that ethnic groups traditionally exposed to malaria have greater frequencies of G6PD deficiency [8,9]. Interestingly, the Africa A mutation was found to be more common among non-UAE citizens than among UAE nationals, indicating that additional genetic diversity influences G6PD deficiency patterns in Abu Dhabi, UAE [5].

Neonatal jaundice is another critical clinical outcome associated with G6PD deficiency. Studies have shown a higher incidence of neonatal jaundice in G6PD-deficient neonates compared to those with normal enzyme activity. This condition results from the inability of the liver to conjugate bilirubin efficiently, leading to its accumulation in the blood. Research indicates that G6PD deficiency exacerbates the risk of severe hyperbilirubinemia, necessitating close monitoring and early intervention to prevent complications such as kernicterus [10]. A study conducted in Nigeria found that G6PD-deficient neonates had a significantly higher likelihood of developing jaundice within the first week of life compared to non-deficient neonates, with jaundice appearing significantly earlier in the deficient group. Additionally, the incidence of kernicterus and mortality was higher among G6PD-deficient neonates [9]. A pilot screening program in the United States found that G6PD-deficient newborns had considerably higher total bilirubin levels and were more likely to need phototherapy than non-deficient infants, highlighting the danger of hyperbilirubinemia in G6PD-deficient neonates [11]. 

G6PD deficiency is a well-recognized cause of neonatal hemolysis and severe hyperbilirubinemia due to increased erythrocyte susceptibility to oxidative stress [12]. Phototherapy remains the primary treatment modality for neonatal jaundice, particularly in hemolytic conditions where bilirubin levels rise rapidly and exceed treatment thresholds early in life [13]. Due to the high frequency of G6PD deficiency in the Middle East, a focused study is required to identify the local variables impacting this condition. Despite the documented genetic tendency, additional contributing variables such as environmental exposures, maternal health, and demographic features within this specific community must be investigated. The goal of this study is to address significant knowledge gaps regarding the genetic, social, and environmental variables impacting this prevalent enzymatic condition.

This study aimed to identify baseline risk factors (such as sex, ethnicity, and family history) associated with G6PD deficiency among neonates admitted to Thumbay University Hospital, Ajman, UAE, and to evaluate their clinical outcomes, including jaundice, bilirubin levels, phototherapy requirement, and length of hospital stay, along with their relationship to G6PD enzyme levels.

## Materials and methods

This study employed a case-control design to identify baseline risk factors and clinical outcomes associated with G6PD deficiency among neonates admitted to Thumbay University Hospital, Ajman, UAE. The case-control approach was appropriate, as it allowed direct comparison between neonates with G6PD deficiency (cases) and those without the condition (controls), facilitating the identification of relevant risk factors. The study was conducted in the neonatal unit, with additional data obtained from outpatient follow-up clinics to ensure comprehensive information on neonates up to one month of age. Cases included neonates with G6PD deficiency confirmed by quantitative laboratory assay during the neonatal period. G6PD deficiency was diagnosed using a quantitative enzymatic assay on blood samples collected within 48-72 hours of life. A cutoff value of < 4.6 U/g Hb was used to define deficiency. Cases were selected consecutively from all neonates who tested positive for G6PD deficiency through the national newborn screening program, regardless of gender. Controls were neonates who tested negative for G6PD deficiency during the same screening process. Controls were selected from neonates who underwent the same newborn screening and were confirmed to be G6PD-normal. Universal newborn screening ensured that all neonates were tested, minimizing selection and ascertainment bias. 

The study period was November 2024 to March 2025. The study population included all neonates born during this period. Exclusion criteria were neonates with known hemolytic disorders other than G6PD deficiency, significant congenital anomalies, lack of parental consent, or incomplete medical records.

The sample size was calculated based on the expected prevalence of baseline risk factors among controls, estimated at 50% from previous regional studies [6]. The “exposure” in this context refers to the presence of at least one baseline risk factor (male sex, positive family history, or specific ethnicity). An odds ratio of 3 was assumed to detect a clinically meaningful difference in risk factor prevalence between cases and controls. Using a 5% significance level, 80% power, and a 1:2 case-to-control ratio, a minimum of 50 cases and 100 controls were required. Data were collected using a structured and expert-validated checklist that captured demographic characteristics, family history, prenatal and perinatal factors, and neonatal health indicators. Baseline variables included sex, ethnicity, and family history of G6PD deficiency. The Institutional Review Board of Gulf Medical University issued approval (approval number: IRB-COM-STD-74-Oct-2024) prior to data collection.

Jaundice was defined based on clinical documentation and/or elevated serum bilirubin levels and was assessed during the neonatal period as recorded in hospital records. Demographic, clinical, and family history data were extracted using the structured checklist. Statistical analyses included chi-square (χ²) tests for categorical variables and the Mann-Whitney U test for continuous variables. Data were analyzed using IBM SPSS Statistics version 29 (IBM Corp., Armonk, NY, USA).

This study is reported in accordance with the Strengthening the Reporting of Observational Studies in Epidemiology (STROBE) guidelines for case-control studies.

## Results

A total of 150 neonates were included in this case-control study, consisting of 50 cases and 100 controls. Among the G6PD-deficient cases, 94% (n=47) were male, and 6% (n=3) were female, indicating a marked predominance of affected males, which was statistically significant (χ²(1) = 17.19, p < 0.001, phi (φ) = 0.34). The control group consisted of 62% (n=62) males and 38% (n=38) females. Ethnicity was categorized into three groups for analysis: Middle East and North Africa (MENA), which included Egyptian, Iranian, Syrian, and other Middle Eastern nationalities; South Asian, consisting of Indian and Pakistani participants; and Others, which comprised Filipino, French, and all remaining nationalities. Ethnic group was significantly associated with G6PD status (χ²(2) = 8.58, p = 0.014, Cramer’s V = 0.24), with South Asian neonates being relatively less frequent among the affected cases.

A positive family history of G6PD deficiency was reported in 16% (n=8) of cases compared with none among controls, showing a strong association with G6PD status (χ²(1) = 16.90, p < 0.001, φ = 0.34). Gestational age was categorized into early term (≤38 weeks) and full term (>38 weeks; 39-40 weeks), with no significant differences observed between the groups (χ²(1) = 0.97, p = 0.325, φ = 0.08). Length of hospitalization was grouped into ≤2 days and >2 days, and G6PD-deficient neonates were significantly more likely to remain admitted for more than two days (χ²(1) = 24.59, p < 0.001, φ = 0.40), with 40% (n=20) of cases compared to 7% (n=7) of controls requiring extended hospitalization. Feeding mode did not differ significantly between groups (χ²(1) = 1.49, p = 0.222, φ = 0.10).

Jaundice was present in 64% (n=32) of G6PD-deficient neonates compared with 15% (n=15) of controls, showing a significant association with G6PD status (χ²(1) = 37.20, p < 0.001, φ = 0.50). More than half of the G6PD-deficient neonates required phototherapy (56%, n=28), compared with only 15% (n=15) of the controls, which was also statistically significant (χ²(1) = 27.40, p < 0.001, φ = 0.43).

Fetal complications were reported in 18% (n=9) of cases versus 9% (n=9) of controls, and maternal complications occurred in 16% (n=8) of cases and 14% (n=14) of controls, neither reaching statistical significance (fetal: χ²(1) = 2.56, p = 0.110, φ = 0.13; maternal: χ²(1) = 0.11, p = 0.744, φ = 0.03). Likewise, mode of delivery did not differ significantly between groups (χ²(1) = 0.26, p = 0.610, φ = 0.04). Details are provided in Table 1.

**Table 1:** Association Between G6PD Status and Sociodemographic and Clinical Variables MENA: Middle East and North Africa; G6PD: Glucose-6-phosphate dehydrogenase

Variable	Group	Control		Case		Total	p-value
		No.	%	No.	%		
Gender	Male	62	62	47	94	109	<0.001
	Female	38	38	3	6	41	
Ethnicity	MENA	39	39	19	38	58	<0.05
	South Asia	58	58	23	46	81	
	Others	3	3	8	16	11	
Family History of G6PD	Yes	--	--	8	16	8	<0.001
	No	100	100	42	84	142	
Gestational group	<=38	30	30	19	38	49	NS
	>38	70	70	31	62	101	
No. of days of hospitalization	<=2 days	93	93	30	60	123	<0.001
	>2 days	7	7	20	40	27	
Feeding Mode	Bottle	20	20	6	12	26	NS
	Breastfeeding	80	80	44	88	124	
Phototherapy Required	Yes	15	15	28	56	43	<0.001
	No	85	85	22	44	107	
Jaundice	Yes	15	15	32	64	47	<0.001
	No	85	85	18	36	103	
Fetal Complications	Yes	9	9	9	18	18	NS
	No	91	91	41	82	132	
Maternal Complications	Yes	14	14	8	16	22	NS
	No	86	86	42	84	128	
Mode of Delivery	Cesarean Section	30	30	13	26	43	NS
	Normal	70	70	37	74	107	

Table 2 presents the comparison of continuous variables between G6PD-deficient cases and controls using non-parametric analysis. Birth weight was lower among cases (median = 3.05 kg, IQR 2.85-3.20 kg) compared to controls (median = 3.30 kg, IQR 3.10-3.55 kg), p = 0.005. Gestational age did not differ significantly (cases: median 39.0 weeks, IQR 38-39 weeks; controls: median 39.0 weeks, IQR 38-40 weeks; p = 0.319). G6PD enzyme levels were markedly lower in cases (median 2.70 U/g Hb, IQR 2.50-3.10 U/g Hb) than in controls (median 9.10 U/g Hb, IQR 8.50-9.50 U/g Hb), p < 0.001. Total serum bilirubin levels were higher in cases (median 13.3 mg/dL, IQR 11.5-15.0 mg/dL) compared with controls (median 8.6 mg/dL, IQR 7.5-9.5 mg/dL), p < 0.001.

**Table 2:** Comparison of Continuous Variables Between G6PD-Deficient Cases and Controls Using Non-parametric Analysis Continuous variables are expressed as median (IQR). The Mann-Whitney U test was used for group comparisons. G6PD: glucose-6-phosphate dehydrogenase

Variable	Control Median (IQR)	Case Median (IQR)	p-value
Birth Weight (kg)	3.30 (3.10–3.55)	3.05 (2.85–3.20)	0.005
Gestational Age (weeks)	39.0 (38–40)	39.0 (38–39)	0.319
G6PD Level (U/g Hb)	9.10 (8.50–9.50)	2.70 (2.50–3.10)	<0.001
Total Bilirubin (mg/dL)	8.6 (7.5–9.5)	13.3 (11.5–15.0)	<0.001

## Discussion

In our study, the observed male predominance among G6PD-deficient neonates is biologically expected, as G6PD deficiency is an X-linked disorder and has been consistently reported to occur more frequently in males across population-based epidemiological studies [7]. Epidemiological studies from India, China, and countries of the Arabian Peninsula similarly demonstrate that male infants constitute the majority of clinically affected neonates, reflecting both genetic transmission patterns and hemizygosity in males [14]. The male bias observed in our cohort, therefore, aligns with established global trends and supports the underlying genetic mechanism of the disorder [15].

Our finding of a significant association between ethnicity and G6PD status, with relatively fewer South Asian neonates among affected cases, aligns with previously documented ethnogeographic differences in G6PD deficiency prevalence. Koromina et al. (2021) found that large population studies have shown that the frequency of G6PD deficiency varies substantially across major ethnic and geographic groups, with the highest rates reported in African and Mediterranean populations and lower prevalence estimates in South Asian populations compared with some other regions (e.g., 2.7%-3.5% in Asia versus 12.2% in Africans) [16].

In our cohort, gestational age did not differ significantly between G6PD‑deficient neonates and controls; similarly, multiple studies have reported no meaningful differences in gestational age distribution between neonates with and without G6PD deficiency, like the study reported by Ullah and Kumar (2025) [17].

Although the mode of delivery did not differ significantly between G6PD‑deficient neonates and controls in our cohort, this aligns with previously published evidence by Nassar et al. (2020) showing no significant association between delivery method and G6PD status or related neonatal outcomes, suggesting that cesarean versus vaginal delivery does not independently influence the risk of G6PD‑related hyperbilirubinemia [18].

In our study, although G6PD‑deficient neonates had lower mean birth weight than controls, previous research has not consistently demonstrated a significant relationship between G6PD status and birth weight. For example, a study by Eghbalian et al. (2021) examining clinical and laboratory characteristics in G6PD‑deficient neonates reported no significant association between birth weight and G6PD deficiency status, suggesting that differences in birth weight may reflect population variation and not a direct effect of enzyme deficiency itself [19].

The observed association between positive family history and G6PD deficiency in our study is consistent with the hereditary nature of the disorder. However, due to the retrospective case-control design and reliance on medical record documentation, ascertainment or documentation bias cannot be excluded, and the strength of this association should be interpreted cautiously [20]. Consanguinity, which is relatively common in certain Middle Eastern communities, further increases the transmission likelihood of G6PD variants [21].

In this study, G6PD-deficient neonates demonstrated significantly higher bilirubin levels and greater phototherapy requirements compared with controls, which aligns with the well-documented role of G6PD deficiency as a major contributor to severe neonatal hyperbilirubinemia [22]. A meta‑analysis by Liu et al. (2015) shows that newborns with G6PD deficiency have a significantly higher risk of hyperbilirubinemia and phototherapy compared with G6PD‑normal neonates; the pooled risk ratio for phototherapy is ≈ 3.01 (95% CI: 2.20-4.12) [23]. G6PD-deficient neonates therefore require closer monitoring during the first days of life to prevent bilirubin accumulation.

The strong association between G6PD deficiency and phototherapy need in our cohort, where 56% of affected neonates required treatment, aligns closely with a large retrospective study by Gopagondanahalli et al. (2022) in which 396 of 681 (58%) G6PD‑deficient infants required phototherapy in the first week of life [24]. Early recognition and treatment are critical, as untreated hyperbilirubinemia in G6PD-deficient infants carries a higher risk of progressing to acute bilirubin encephalopathy compared to enzyme-normal neonates [25].

The significantly longer duration of hospitalization observed among G6PD-deficient neonates in this study is consistent with a previous report by Eissa et al. (2021), indicating that affected infants often require prolonged inpatient monitoring [26]. This finding reflects the added clinical burden associated with G6PD deficiency and highlights the importance of extended observation in these neonates.

Our study has several limitations. The sample size, while sufficient for preliminary analysis, may limit generalizability. Selection bias may be present due to retrospective case identification, and reliance on medical records for family history, bilirubin levels, and phototherapy could introduce documentation bias. The exact timing of jaundice onset and phototherapy initiation was inconsistently recorded. Prolonged hospital stay (>2 days) was not adjusted for confounders such as mode of delivery, and no multivariable adjustment was performed, limiting control of potential confounders. Finally, as a single-center study, these findings may not be generalizable to other populations. Further large-scale, multicenter studies with standardized data collection and multivariable analysis are needed to validate these findings.

## Conclusions

G6PD-deficient neonates demonstrated higher rates of jaundice, elevated bilirubin levels, and increased need for phototherapy, highlighting the role of the disorder in severe neonatal hyperbilirubinemia. Male predominance and positive family history were significantly associated, consistent with the genetic basis of the condition. While gestational age, maternal complications, and mode of delivery were not associated, affected neonates had longer hospital stays, reflecting increased clinical burden. These findings emphasize the importance of early screening, close monitoring, and timely intervention, particularly in high-prevalence regions, to reduce morbidity and prevent complications such as bilirubin encephalopathy and kernicterus.
